# An orally active immune adjuvant prepared from cones of *Pinus sylvestris*, enhances the proliferative phase of a primary T cell response

**DOI:** 10.1186/1472-6882-14-163

**Published:** 2014-05-22

**Authors:** William Guy Bradley, Katharine Nichole Holm, Akiko Tanaka

**Affiliations:** 1 Tampa Bay Research Institute, 10900 Roosevelt Blvd N, St. Petersburg, FL 33716, USA; 2 Eckerd College, 4200 54th Ave South, St. Petersburg, FL 33711, USA

**Keywords:** Polyphenylpropanoid polysaccharide complex, Primary T cell response, Pine cone extract

## Abstract

**Background:**

We have previously demonstrated that an alkaline extract of shredded pinecones yields a polyphenylpropanoid polysaccharide complex (PPC) that functions as an orally active immune adjuvant. Specifically, oral PPC can boost the number of antigen-specific memory CD8^+^ T cells generated in response to a variety of vaccine types (DNA, protein, and dendritic cell) and bias the response towards one that is predominately a T helper 1 type.

**Methods:**

An immune response was initiated by intraperitoneal injection of mice with *Staphylococcus* enterotoxin B (SEB). A group of mice received PPC by gavage three times per day on Days 0 and 1. The draining lymph nodes were analyzed 48–96 h post-injection for the numbers of reactive T cells, cytokine production, the generation of reactive oxygen species, and apoptotsis.

**Results:**

In this study we examined whether the ability of PPC to boost a T cell response is due to an effect on the proliferative or contraction phases, or both, of the primary response. We present data to demonstrate that oral PPC significantly enhances the primary T cell response by affecting the expansion of T cells (both CD4 and CD8) during the proliferative phase, while having no apparent effects on the activation-induced cell death associated with the contraction phase.

**Conclusions:**

These findings suggest that PPC could potentially be utilized to enhance the T cell response generated by a variety of prophylactic and therapeutic vaccines designed to target a cellular response.

## Background

The health benefits of a pinecone extract were first documented by Pedanius Dioscorides [1]. Dioscorides (ca. 40-ca. 90) was an ancient Greek physician, pharmacologist and botanist who practiced in ancient Rome. In about AD 65, after much direct observation of plants in their native habitats and careful practical experience on the medicinal uses of herbs, he wrote *De Materia Medica. De Materia Medica* is a precursor to all modern pharmacopeias and is one of the most influential herbal books in history. Within the *Materia Medica*, Dioscorides describes using extracts of pinecones to treat kidney and digestive ailments and to apply to the skin to treat a disease that appears to have been psoriasis.

For much of the last century, the Japanese inhabitants of the island of Kyushu have also known about the medicinal properties of a pinecone extract [2]. They have used an aqueous extract (tea) prepared from pinecones to treat illnesses ranging from infectious disease to cancer. Because of the indigenous beliefs that the pinecone tea was able to treat such a wide variety of illnesses, it appeared to us that the effects of the extract were very likely being mediated through the immune system.

Previous studies indicate that an orally active polyphenylpropanoid polysaccharide complex (PPC) derived from the cones of *Pinus sylvestris* not only biases the immune response initiated by DNA, protein, or dendritic cell vaccines towards one that is predominately a T helper type 1 (Th1), it also significantly enhances the generation of antigen-specific CD8^+^ cytotoxic T cells detected during a secondary T cell response [3]. *In vitro* studies have revealed that the exposure of human PBMC [4] or murine bone marrow cells [5] to PPC rapidly induces the production of dendritic-like cells.

The ability to induce a predominant Th1 response when delivered at the time of immunization is coupled with the ability to suppress the development of an antigen-specific Th2 response [3]. Oral delivery of PPC at the time of immunization with the model Th2 antigen, chicken egg ovalbumin (OVA), blocks the development of an IgE-mediate allergic response to OVA. *In vitro* this is detected as an inability of OVA-stimulated splenocytes to produce IL-4 while producing significantly elevated amounts of IFNγ [3]. When the levels of OVA-specific CD8^+^/IFNγ^+^ T cells in these mice are measured by ELISPOT, the mice receiving PPC at the time of immunization consistently yield twice as many of these cells [3].

With the continual accumulation of anecdotal reports suggesting that use of PPC along with traditional anti-cancer therapies somehow enhances the ability to mount an effective anti-cancer response, we are motivated to better understand PPC's mechanism of action. In the study described herein, we determined that PPC’s association with an increased number of antigen-specific CD8^+^ CTL activated during a secondary T cell response is very likely due to its ability to significantly enhance the expansion of these cells during the early phase of the primary T cell response and not by affecting the contraction phase or rate of activated induced cell death.

## Methods

### Animal care

Six to seven week old male Balb/c mice were obtained from Charles River Laboratories and housed 3–5 mice per cage in a self-contained ventilated cage system (Innovive Inc., San Diego,CA) maintained at 40 air changes per hour. Both the intake and exhaust air were HEPA filtered. Mice were maintained in an environment of 22˚C with a 12 h light/12 h dark cycle. The mice were fed *ad libitum* a standard diet containing 22% crude protein and 5% fat (Harlan Teklad Laboratory, cat no 8640) and provided water *ad libitum*. This study was carried out in strict accordance with the recommendations in the Guide for the Care and Use of Laboratory Animals of the National Institutes of Health. The protocol was approved by the Tampa Bay Research Institutional Animal Care and Use Committee (protocol number: 2011–001).

### Preparation of PPC

Powdered PPC was kindly provided by Allera Health Products, Inc. (360 Central Avenue, Suite 1560, St. Petersburg, FL 33701). The pinecones used by Allera to produce PPC were obtained from a commercial supplier of Scotch pine (*Pinus sylvestris*) (identification of the supplier is regarded as proprietary information). Identification of the specific species of pine was performed by a qualified employee of the supplier. The pinecones were washed extensively in deionized water and then shredded. The pinecone shreds were then loaded into a stainless steel reaction vessel and extracted with water at elevated pH (12.0) and temperature (121°C, 30 min). The resulting extract was standardized by UV spectroscopy at a wavelength of 280 nm and by a series of biological assays. The liquid was tested extensively to ensure that it did not contain contaminants such as herbicides, pesticides, heavy metals, endotoxin, or microorganisms and was then spray-dried to form a dark brown, odorless, stable powder. The powdered extract was suspended in sterile water to a stock concentration of 25 mg/mL (w/v), centrifuged at 10,000 × g for 20 minutes to remove particulates and then filtered through a 0.2 μm nylon filter. The OD_280_ of the solution was measured and used to define the concentration of active ingredients:

$$\text{PPC}\;\text{conc}\;\left(\mathrm{mg}/\mathrm{mL}\right)=OD_{280}\times \;\left(1/22.8\;L\times \text{gra}m^{-1}\times cm^{-1}\right)$$

where 22.8 L⋅gram^-1^⋅cm^-1^ is the extinction coefficient for polyphenylpropanoids. A working stock containing 200 μg/mL of polyphenylpropanoids diluted in sterile distilled water was prepared fresh each week.

### *In Vivo* immune stimulation

On Day 0, three mice each in the SEB and SEB + PPC groups were injected intraperitoneally (i.p.) with 100 μg of Staphylococcal enterotoxin B (SEB, Sigma Chemical Company, St. Louis, MO) dissolved in 100 μL phosphate buffered saline (PBS), pH 7.4. Three mice in the Naive group were injected i.p. with 100 μL PBS, pH 7.4. The mice in the SEB + PPC group were gavaged with 100 μL of a 200 μg/mL solution of PPC three times daily on Day 0 and Day 1 for a total of 6 doses. At 48 or 96 hours post-SEB immunization, the mice were euthanized by CO_2_ inhalation. The set of inguinal lymph nodes from each mouse was pooled and used in each assay. Therefore, the results reported from lymph node cells represent the collection of data points from each mouse. The number of cell subsets per lymph node was obtained from the set of pooled inguinal lymph nodes and then dividing that number by two. To calculate the total number of cell subsets per lymph node, the percent of each subset was multiplied by the total number of cells isolated from the lymph nodes. The total number of cells isolated from the lymph nodes was determined using the Millipore Muse cell counter. Serum was collected for quantification of various cytokines and was stored at -80°C until analyzed.

### ELISA assays

Standard ELISA development kits specific for murine IL-2, IFNγ, and IL-12p70 were purchased from Peprotech (Rocky Hill, NJ). The detection of serum IL-2, IFNγ, and IL-12p70 was performed according to the manufacturer’s instructions. The absorbance of the final product was measured using BioTek’s μQuant plate spectrophotometer at a wavelength of 450 nm. Standard curves were prepared using Prism’s GraphPad software (GraphPad Software, Inc., La Jolla, CA).

### Lymph node and spleen cell isolation

Isolated inguinal lymph nodes and spleens were homogenized in a sterile plastic bag containing 4 mL Hanks balanced salt solution (HBSS) pH 7.4, using a Seward Stomacher^®^80 with settings of medium speed for 60 seconds. The resulting cell suspensions were pelleted and then suspended in 2 mL HBSS (for lymph node cells) or 4 mL HBSS (for spleen cells). The yield of cells was determined by mixing 20 μL cells with 380 μL of Millipore’s Muse Count and Viability stain. The cell concentration and number of viable cells was determined using the Muse Cell Analyzer (Millipore). The cells were pelleted and then suspended in enough complete media (RPMI 1640 containing 10% fetal calf serum and 1x penicillin/streptomycin) to provide a final concentration of 4 × 10^6^ cells/mL.

### Flow cytometry

For each mouse, 1×10^6^ lymph node cells were added to a 1.5 mL microfuge tube and then pelleted by centrifugation for 1 minute at 1000 × g. The pelleted cells were placed on ice and then suspended in 100 μL of FACS buffer (PBS containing 10% fetal bovine serum and 0.1% sodium azide) containing 1 μL anti-CD16/32 Fc blocking antibody (clone 93). Ten minutes later 1–5 μL of fluorescent antibodies specific for cell surface antigens were added. The fluorescent anti-mouse antibodies used to detect the SEB-activated T lymphocytes were as follows: CD3 (clone 17A2) labeled with allophycocyanin (APC), CD4 (clone GK1.5) labeled with fluorescein isothiocyanate (FITC), CD8 (clone SK1) labeled with phycoerythrin (PE), and Vβ8 (clone KJ16-133) labeled with a proprietary peridinin chlorophyll protein conjugated with eFluor710 (PerCP-eFluor^®^710). All of the antibodies were purchased from eBioscience, San Diego, CA. The cells were incubated on ice for 30 min and then washed by adding 500 μL FACS buffer and pelleting the cells at 1000 × g for 2 min. The pelleted cells were then suspended in 200 μL FACS buffer and analyzed by flow cytometry using a BD FACSCalibur (Becton Dickinson, San Jose, CA) equipped with CellQuest Pro software. The FACS data was further analyzed using FloJo software (TreeStar Inc, Ashland, CA).

### Detection of apoptosis

Annexin V staining was performed according to the protocol provided with eBioscience’s Annexin V Apoptosis Detection Kit. Isolated lymph node cells (1 × 10^6^/mouse) were suspended in Annexin V staining buffer containing 1 μL APC-labeled anti-CD3, 5 μL PerCP-eFluor^®^710-labeled anti-Vβ8, and 1 μL FITC-labeled Annexin V to identify T cells undergoing apoptosis. The cells were incubated at room temperature for 10 min and then washed in binding buffer. The cells were suspended in binding buffer and then analyzed by flow cytometry.

### Detection of reactive oxygen species

To detect the presence of reactive oxygen species in activated T cells, isolated lymph node cells from each mouse were exposed to Invitrogen’s CM-H_2_DCFDA (5-(and-6)-chloromethyl-2’ ,7’-dichlorodihydrofluorescein diacetate, acetyl ester). CM-H_2_DCFDA passively diffuses into cells where its acetate groups are cleaved by intracellular esterases and its thiol-reactive chloromethyl group reacts with intracellular glutathione and other thiols. Oxidation then produces a fluorescent adduct that cannot escape the cell. The resulting fluorescence was then measured by flow cytometry. Isolated lymph node cells (1 × 10^6^/mouse) were exposed to 100 μL FACS buffer containing 1 μL APC-labeled anti-CD3 antibody, 5 μL PerCPeFluor^®^710-labeled anti-Vβ8 antibody and 2 μL of a 50 μM solution of CM-H_2_DCFA for 15 min at 37˚C. The cells were then washed with 500 μL FACS buffer, pelleted and suspended in 200 μL FACS buffer. The levels of fluorescence within the various cell samples were measured by flow cytometry. The resulting data is shown as the percentage of ROS^+^ lymph node T cells (CD3^+^) and as the frequency distribution (histogram) of ROS levels in lymph node CD3^+^ T cells in SEB and SEB + PPC treated mice.

### Detection of intracellular Bcl-2

To detect the intracellular levels of the anti-apoptosis related protein, Bcl-2, cells previously stained with APC-labeled anti-CD3 and PerCPeFluor^®^710-labeled anti-Vβ8 antibodies were washed in 0.03% saponin dissolved in HBSS (HBSS-SAP) and then pelleted by centrifugation at 1000 × g for 2 minutes. The cell pellets were suspended in 100 μL HBSS-SAP containing 1 μL FITC-labeled anti-Bcl-2 antibody (clone 10C4, eBioscience), and incubated on ice for 60 minutes. The cells were washed twice with 500 μL HBSS-SAP buffer and then once with HBSS only. The cells were then suspended in 200 μL HBSS and analyzed by flow cytometery.

### Analysis of gene expression

Total RNA was isolated from lymph node cells using Qiagen’s RNeasy Plus Mini Kit following the manufacturer’s instructions. The resulting RNA was eluted in 30 μL sterile RNase-free water and the yield of RNA was determined by spectrophotometry at an optical density of 260 nm.

One microgram of total RNA was reverse-transcribed into cDNA using the QuantiTect Reverse Transcription kit from Qiagen (Valencia, CA) and by following the manufacturer’s instructions. SABiosciences’ (Valencia, CA) RT^2^ Profiler qPCR SYBR green master mix kit was used for the real time quantitative PCR (qPCR). One microliter of the cDNA per reaction was added to a 1.5 mL microfuge tube containing 12.5 μL 2x SYBR green/fluorescein master mix and 10.5 μL H_2_O per reaction needed. Validated primer pairs from SABiosciences' RT^2^ qPCR Primer Assays specific for murine Bcl-2, Bcl-3, Bcl-xL, Bax, and Bim were utilized. These primers are pre-mixed at a concentration of 10 μM. One microliter of each primer pair was placed in duplicate wells of a 96 well PCR plate and then 24 μL of the cDNA/SYBR green master mix was added to the appropriate wells. Once all the samples had been added to the plate the plate was covered with clear plastic film and then centrifuged at 200 × g for 1 min to mix and collect all of the solutions in the bottom of the wells. The plate was then placed in a BioRad (Hercules, CA) iCycler programmed to run one cycle of 10 min at 95˚C to activate the polymerase and then 40 cycles of [95˚C × 15 sec → 60˚C × 1 min] to amplify the gene-specific cDNA. The relative levels of mRNA expression were normalized to the mouse β-actin housekeeping gene expressed in lymph node cells from naive (untreated) mice and determined by the ∆∆-Ct method [6].

### Statistical analysis

The significance of responses to the multiple treatments was determined by ANOVA using Newman-Keuls multiple comparison test provided in GraphPad’s Prism^®^ software (GraphPad Software, Inc, La Jolla, CA). All experiments were performed at least 3 times. Error bars on the graphs represent the standard deviation and demonstrate the ability to detect statistical significance when using only 3 mice per group.

## Results

### Oral delivery of PPC enhances the number of CD4^+^/ and CD8^+^/Vβ8^+^ T cells in the lymph nodes of mice during the primary response to SEB

**Figure 1:**
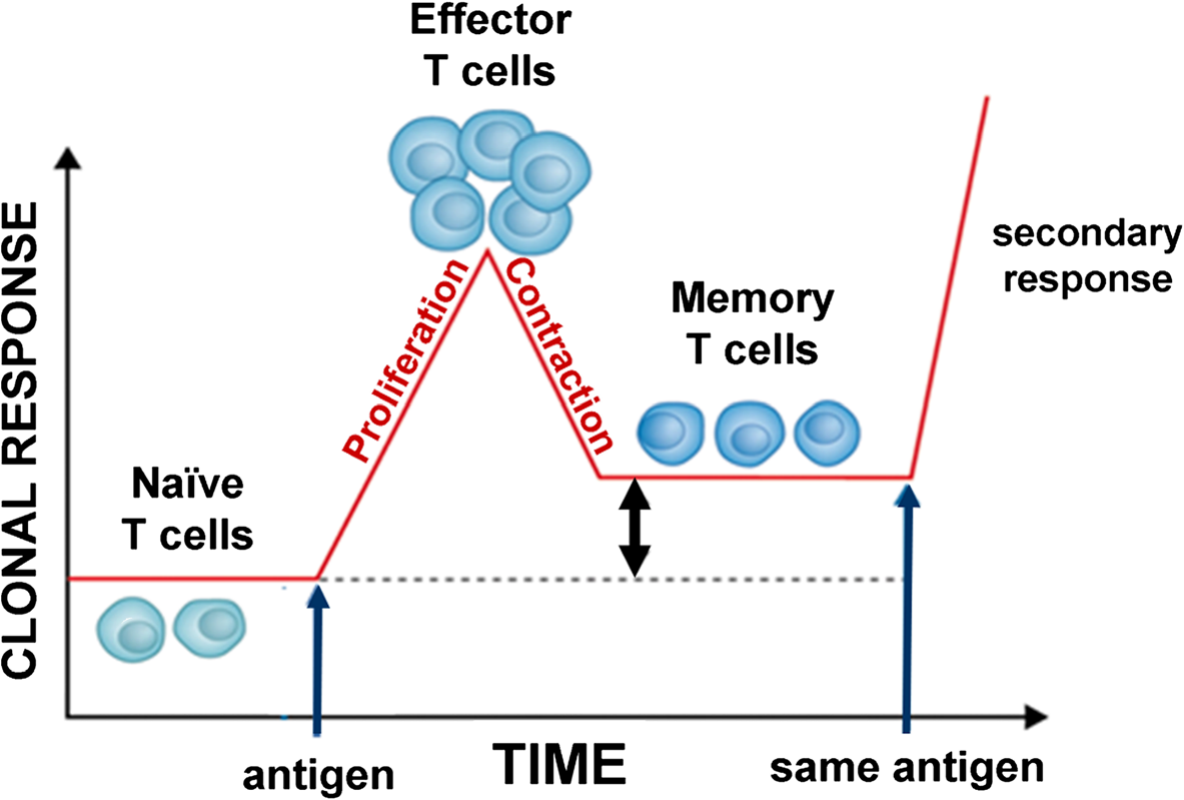
**Kinetics of the primary T cell response. **In the primary response to antigen, naive T cells undergo a massive proliferative burst of expansion that creates short-lived effector cells. This proliferative phase is then followed by a contraction phase in which the majority (90–95%) of the antigen-specific T cells undergo apoptosis, leaving behind a stable memory population.

Previous studies have shown that the injection of SEB specifically induces the proliferation of murine CD3^+^/Vβ8^+^ T cells in a dose-dependent manner during a primary T cell response (Figure 1) [7]. To determine if PPC would enhance this proliferative response to SEB, mice (3 per group) were divided into four experimental groups. While the naive groups received no treatment, the SEB group received an i.p. injection of 100 μg SEB on Day 0, and the SEB + PPC group received an i.p. injection of SEB on Day 0 that was immediately followed by gavage with 100 μL of a 200 μg/mL solution of PPC. This gavage was repeated two more times on Day 0 and then three times (10 am, 1 pm, and 4 pm) on Day 1. Starting on Day 0 the mice were also supplied 200 μg/mL PPC in their water *ad libitum*. PPC was also supplied in this manner to the PPC only group of mice. This experimental protocol was repeated several times for durations of 48 hours to determine if PPC affected cell activity during the proliferation phase of the primary T cell response.

**Figure 2:**
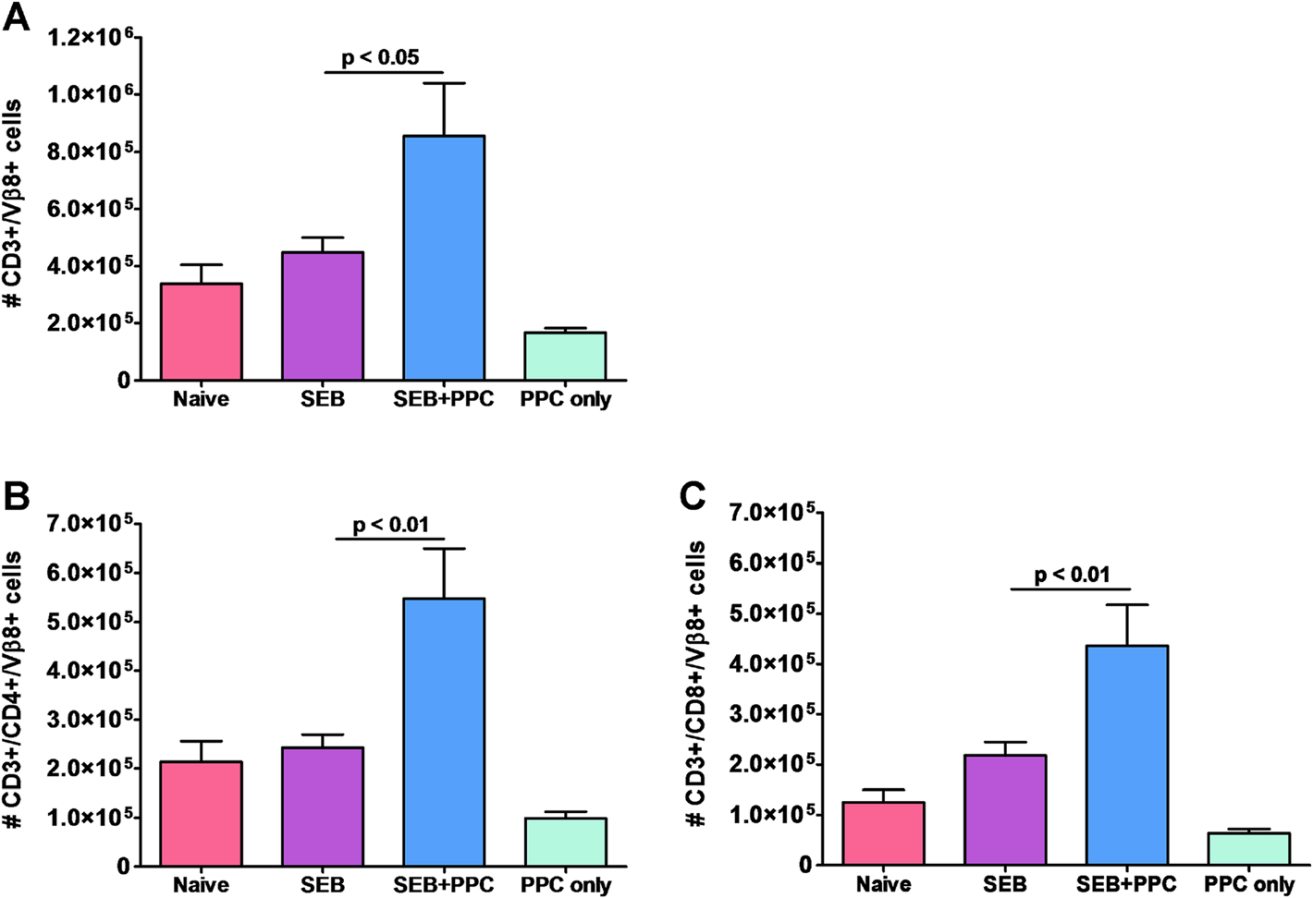
**Expansion of Vβ8**^**+ **^**T cells in SEB + PPC treated mice after 48 hours.** Balb/c mice (n = 3 per group) were injected i.p. with 100 μg SEB only (SEB), gavaged with 100 μL PPC at a concentration of 200 μg/mL (PPC only) or injected i.p. with 100 μg SEB and gavaged with 100 μL of PPC at 200 μg/ml TID for 2 days (SEB + PPC). The SEB + PPC and PPC only mice were also provided PPC at 200 μg/mL in their drinking water *ad libitum* for 48 hours. Naive mice received an i.p. injection of PBS. **(A)** The total number of CD3^+^/Vβ8^+^ cells, **(B)** CD3^+^/CD4^+^/Vβ8^+^ cells, and **(C)** CD3^+^/CD8^+^/Vβ8^+^ cells per lymph node are shown. Error bars represent standard deviation.

At the height of T cell proliferation, 48 hours post SEB injection, significantly more CD3^+^/Vβ8^+^ T cells were isolated from the inguinal lymph nodes of mice treated with SEB + PPC (Figure 2A, p < 0.05). This PPC associated enhancement of lymph node T cells was detected in both the CD4^+^/Vβ8^+^ (p < 0.01) and CD8^+^/Vβ8^+^ populations (p < 0.01) (Figure 2B and 2C, respectively). Interestingly, administration of PPC alone had no effect on the numbers of CD3^+^/, CD4^+^/, or CD8^+^/Vβ8^+^ T cells (p > 0.05 for all three populations of Vβ8^+^ T cells when comparing Naïve versus PPC only) (Figure 2).

PPC enhances the number of CD8^+^/Vβ8^+^ T cells surviving the contraction phase of the primary T cell response

**Figure 3:**
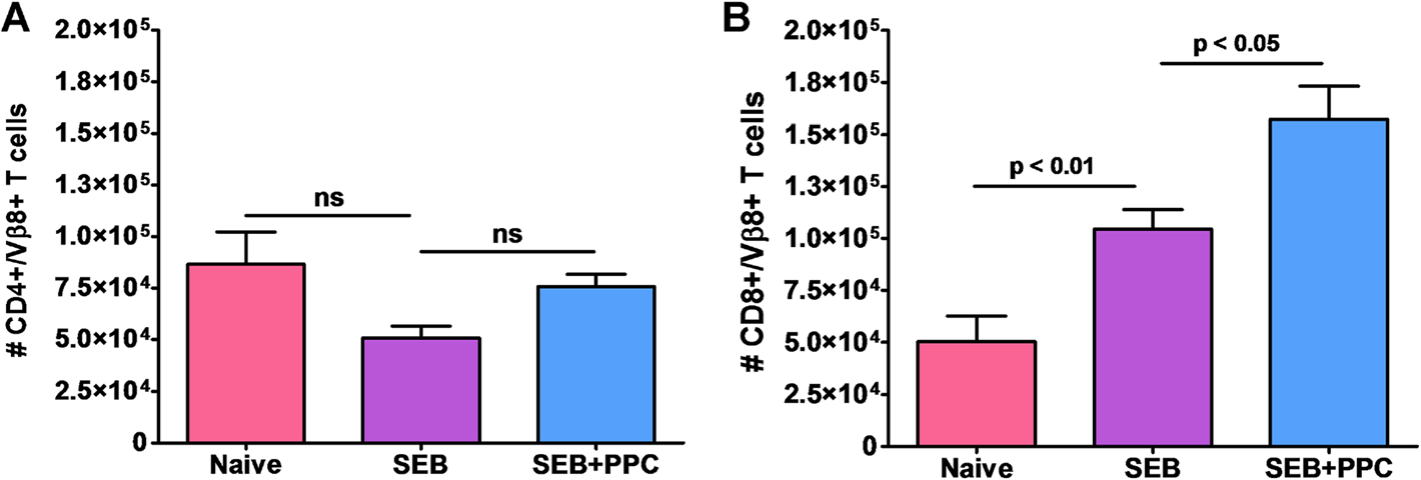
**Levels of Vβ8**^**+ **^**T cells in the inguinal lymph nodes 96 h post SEB injection.** Balb/c mice (n = 3 per group) were treated as described in the Materials and Methods. **(A)** The total number of CD4^+^/Vβ8^+^ cells and **(B)** CD3^+^/CD8^+^/Vβ8^+^ cells per lymph node are shown. Error bars represent standard deviation.

Separate groups of mice were treated as described above and then at the peak of the contraction phase (Figure 1), 96 h post i.p. injection of 100 μg SEB, the inguinal lymph node Vβ8^+^ T cells were enumerated by flow cytometry to determine if oral delivery of PPC affected this phase of the primary response. As shown in Figure 3 (left panel), by 96 h the numbers of CD3^+^/CD4^+^/Vβ8^+^ T cells in the SEB and SEB + PPC groups had contracted to levels no different than that found in the Naïve group (p > 0.05). However, the numbers of CD3^+^/CD8^+^/Vβ8^+^ T-cells remained elevated in both the SEB (Figure 3, right panel; p < 0.01 compared to the Naïve group) and SEB + PPC treated groups (p < 0.05 when compared to the SEB group). This seemingly enhanced susceptibility of CD4^+^ cells to activation induced cell death (AICD) has been reported by others and is not specific for the response to superantigens [8,9]. As observed in the 48 h samples, treatment with PPC was associated with a significant increase in CD8^+^/Vβ8^+^ T cells when compared to the samples from mice receiving only the SEB injection (p < 0.05, Figure 3, right panel).

### PPC reduces serum IL-2 and enhances IFNγ levels 48 h post immunization

**Figure 4:**
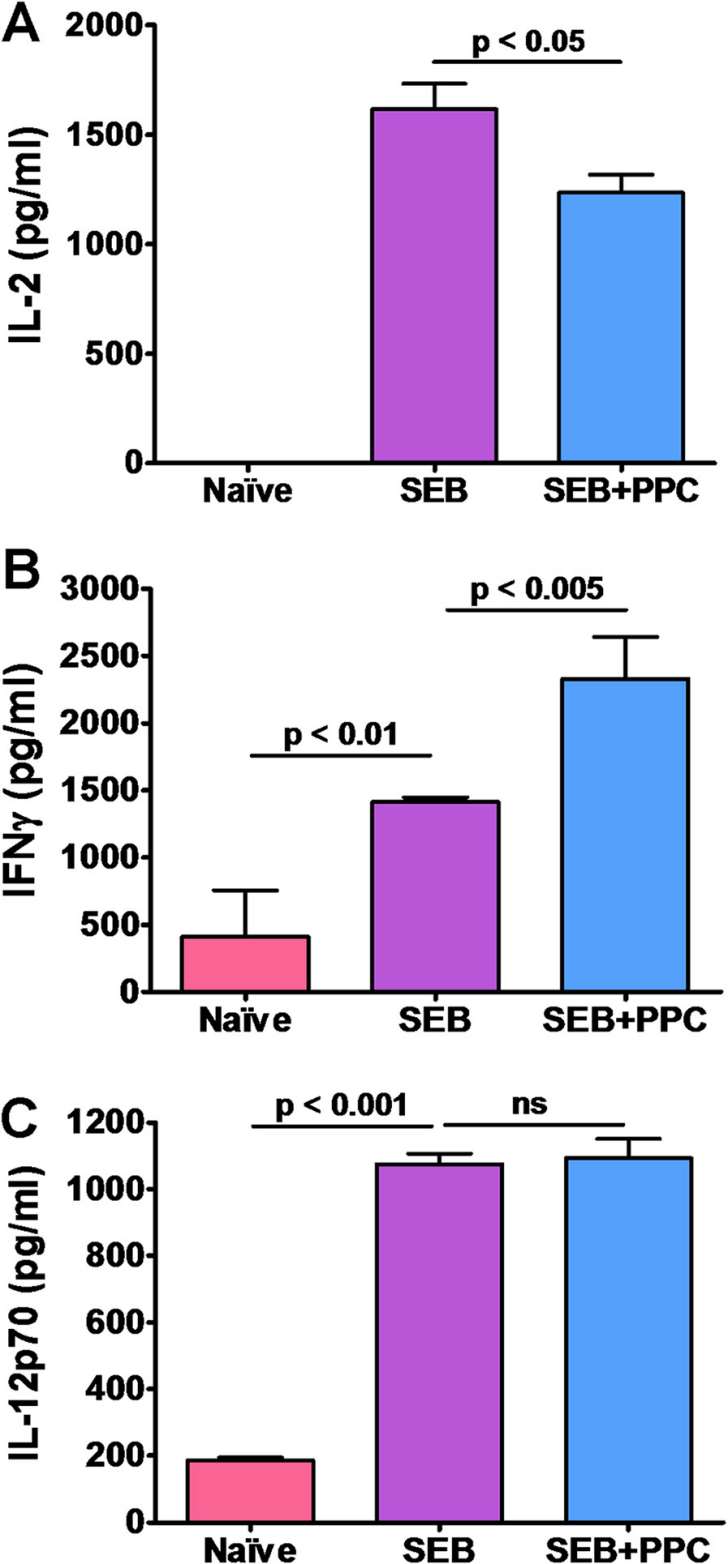
**Serum cytokine levels in mice 48 h post i.p. injection of SEB.** Mice were injected i.p. with 100 μg SEB. One group was provided PPC (200 μg/mL) in their drinking water for 48 h (blue bars). Naive mice received an i.p. injection of PBS (red bars). After 48 h serum was obtained and examined by ELISA for the levels of IL-2 (panel **A**), IFNγ (panel **B**), and IL-12p70 (panel **C**). Error bars represent standard deviation.

To determine if oral administration of PPC was affecting the production of cytokines associated with T cell activation and expansion, we examined the serum from each mouse in the various treatment groups for the presence of IL-2, IFNγ, and IL-12p70. The results reveal that immunization with SEB resulted in the significant increase in the serum levels of all three cytokines (Figure 4). The serum from mice receiving PPC along with the SEB immunization were found to have less IL-2 (p < 0.05), more IFNγ (p < 0.005), and no significant change in the levels of IL-12p70 (Figure 4).

### PPC reduces the levels of ROS in SEB activated CD3^+^ lymph node T cells 48 h post immunization

**Figure 5:**
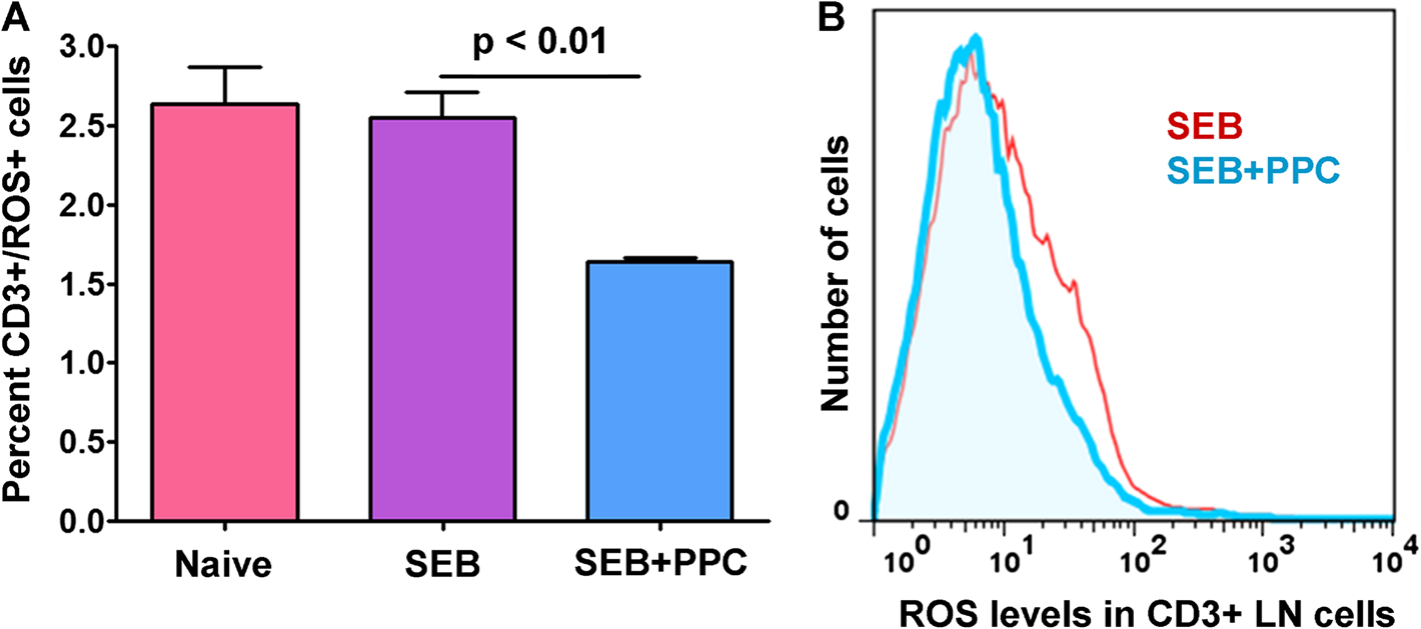
**ROS levels in CD3**^+ ^**lymph node T cells 48 h post SEB injection.** Balb/c mice (n = 3 per group) were treated as described in the Materials and Methods. **(A)** The percentage of CD3^+^/ROS^+^ lymph node T cells from each treatment group are shown. **(B)** The effect of PPC on the level of ROS per CD3^+^ lymph node T cell in the SEB (red line) and SEB + PPC groups (blue line) are shown. Error bars represent standard deviation.

Since the increase in reactive oxygen species (ROS) produced by activated T cells is necessary for the induction of apoptosis during the contraction phase of the primary T cell response [10], the levels of ROS in the lymph node T cells from each of the treatment groups were determined. As shown in Figure 5 (left panel), oral delivery of PPC significantly reduced the percentage of CD3^+^/ROS^+^ T cells obtained from SEB treated mice (p < 0.01) and significantly reduced the levels of ROS per individual CD3^+^ T cells (Figure 5, right panel). This would suggest that molecules in PPC might be functioning as antioxidants *in vivo*. If this is true, PPC could be affecting the ability of ROS to induce apoptosis in the activated T cells. For these reasons the expression of several apoptosis related genes was investigated.

### PPC’s effect on the expression of the anti-apoptotic protein, Bcl-2, 48 hr post immunization

**Figure 6:**
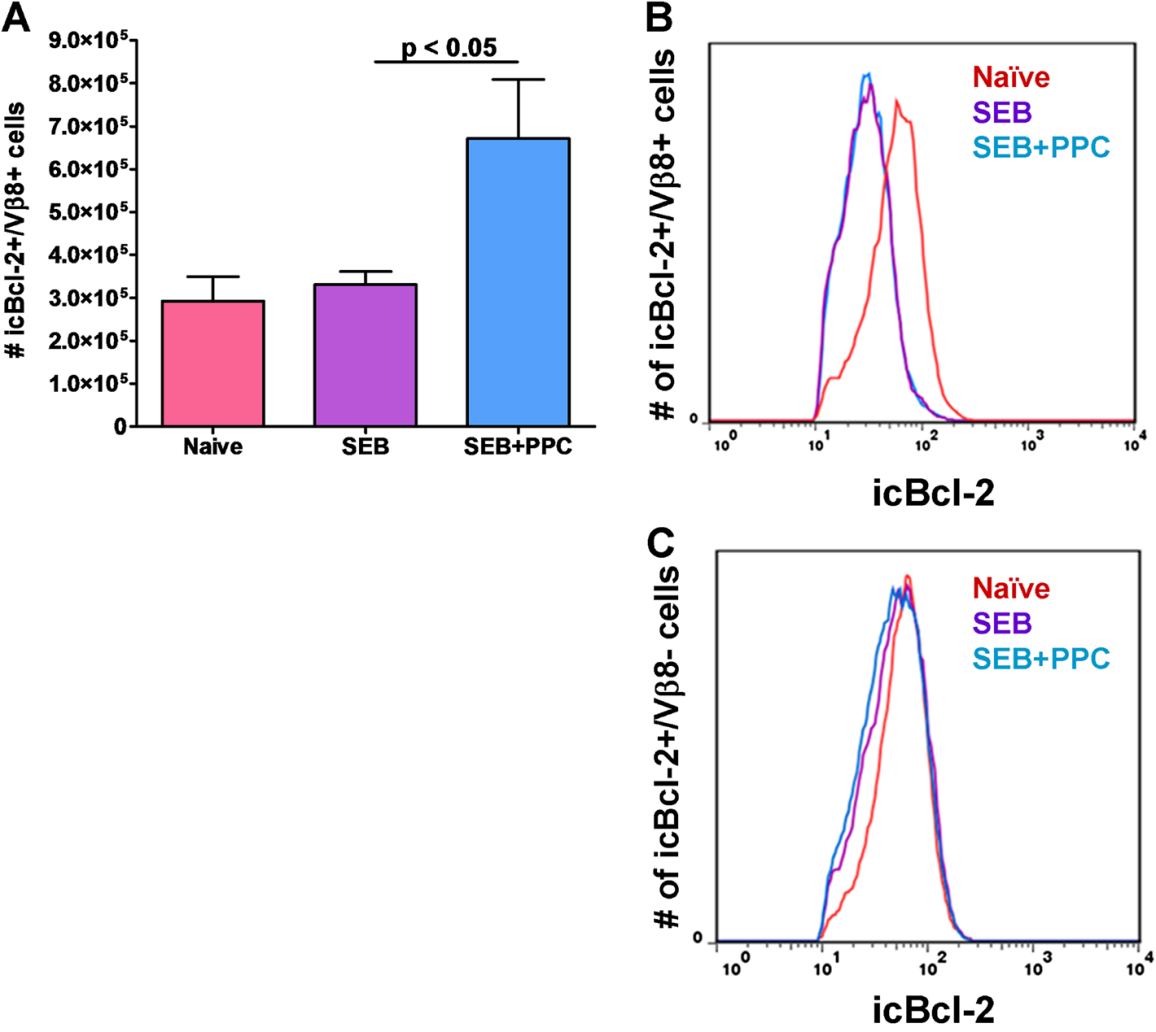
**Intracellular levels of Bcl**-**2 in Vβ8**^**+ **^**lymph node T cells 48 h post SEB injection.** Balb/c mice (n = 3 per group) were treated as described in the Materials and Methods. The number of intracellular Bcl-2^+^/Vβ8^+^ lymph node T cells from the Naive (red line), SEB (purple line), and SEB + PPC groups (blue line) are shown in the bar graph (panel **A**) and the histogram in panel **B**. Error bars in panel A represent standard deviation. The histogram in panel **C** depicts the number of intracellular Bcl-2^+^/Vβ8^-^ cells detected in the lymph nodes from the different treatment groups.

**Figure 7:**
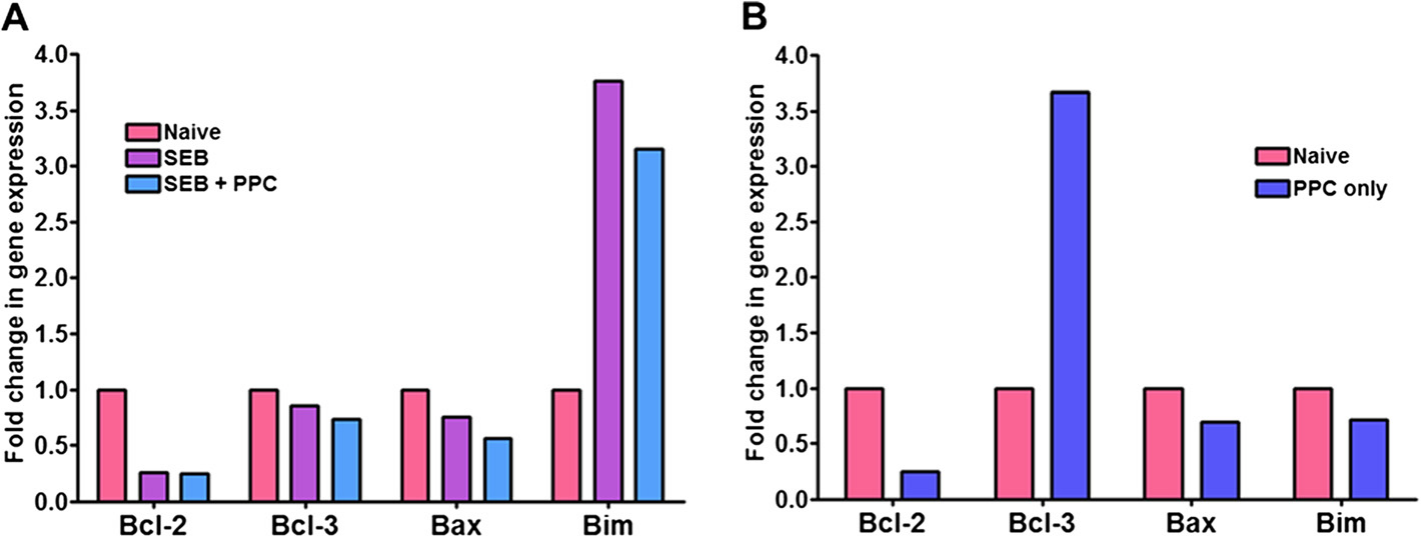
**Expression of Bcl**-**2**, **Bcl**-**3**, **Bax and Bim mRNA in lymph nodes 48 h post SEB injection.** Balb/c mice (n = 3 per group) were treated as described in the Materials and Methods. **(A)** mRNA from naïve, SEB or SEB + PPC treated mice was analyzed for the expression of Bcl-2, Bcl-3, Bax, and Bim, while **(B)** expression of the same genes in mice treated with PPC only were analyzed. Total cell RNA was pooled from the lymph nodes of Naïve, SEB and SEB + PPC treated mice and then converted to ssDNA. Duplicate samples from each group were used for RT-PCR. The fold change in RNA levels for each gene was determined using the ∆∆-Ct method and β-actin as the expression control.

Hildeman et al. revealed that the ROS production by T cells activated with SEB results in the suppression of Bcl-2 mRNA and protein expression [11]. The suppression of Bcl-2 expression reduces the Bcl-2/Bim ratio and allows unbound Bim to interact with Bax and create the apoptosome [12]. If T cells are activated in the presence of an antioxidant the levels of Bcl-2 mRNA and protein increase and lead to significant inhibition of ACID [13]. Since PPC appeared to have reduced the levels of ROS in SEB activated T cells, we sought to determine if this was also associated with a concomitant increase in Bcl-2 mRNA or protein expression. While PPC appeared to enhance the percentage of CD3^+^/Vβ8^+^ T cells positive for the intracellular expression Bcl-2 protein (p < 0.05; Figure 6A), this was found to be due to the increased number of CD3^+^/Vβ8^+^ T cells in the PPC-treated mice rather than an increase in the level of Bcl-2 protein per cell (Figure 6B). When individual Vβ8^+^ T cells were examined, the T cells from mice in both the SEB and SEB + PPC groups contained equally reduced amounts of intracellular Bcl-2 protein (Figure 6B). Bcl-2 levels in Vβ8 negative cells appeared not to be affected by SEB or SEB + PPC (Figure 6C). These results suggest that the reduction of ROS levels detected in the SEB + PPC-treated mice was not likely to have affected the induction of apoptosis.The reduction of Bcl-2 protein levels in the SEB and SEB + PPC groups was paralleled by a reduction in the levels of Bcl-2 specific mRNA detected in the lymph nodes (Figure 7A). The treatment of naïve mice with PPC only also resulted in a reduction in the expression level of Bcl-2 mRNA. This would suggest that PPC most likely does not function as an antioxidant in vivo and exerts little effect on the pattern of activation induced apoptosis.

### PPC’s effect on the expression of mRNA for Bcl-3, Bim, and Bax

Overexpression of the NFkB activator, Bcl-3, is associated with enhanced survival of activated T cells. Cytokines from adjuvant activated dendritic cells enhances the expression of Bcl-3 mRNA [14]. In the SEB and SEB + PPC treated mice we observed no significant effect of PPC on the levels of the Bcl-3 mRNA (Figure 7A). This also suggested that PPC was not affecting the expression of anti-apoptosis genes.

However, there was observed an almost 4-fold increase in Bcl-3 mRNA in the lymph nodes of mice treated with only PPC (Figure 7B). In the study by Mitchell et al. [15] neither Bcl-2 nor Bcl-xL expression in T cells was found to be correlated with activation induced survival (AIS). In their paper further analysis showed that neither the extent of T cell proliferation nor the reactive oxygen content of T cells was affected in a way that could explain AIS. Finally, they showed that microarray analyses pointed toward the expression of Bcl-3 as an important mediator of AIS in T cells [15]. Our results suggest that while PPC can enhance expression of Bcl-3 mRNA in naïve mice, it apparently cannot influence Bcl-3 expression in SEB-activated T cells.While the mRNA levels for the pro-apoptosis protein, Bax, did not appear to be significantly affected by SEB or SEB + PPC treatment (Figure 7A), the levels of mRNA for the pro-apoptosis Bcl-2 binding protein, Bim, were found to be elevated almost 4-fold in the lymph nodes from mice 48 h after treatment with SEB or SEB + PPC (Figure 7A). The administration of PPC alone did not appear to affect the expression of Bim (Figure 7B). These observations support the conclusion that the pattern of AICD is clearly detected in the SEB treated mice and that administration of PPC to these mice does not appear to significantly suppress the activation induced apoptosis associated with the contraction phase of T cell activation.

### PPC does not suppress SEB-enhanced apoptosis

**Figure 8:**
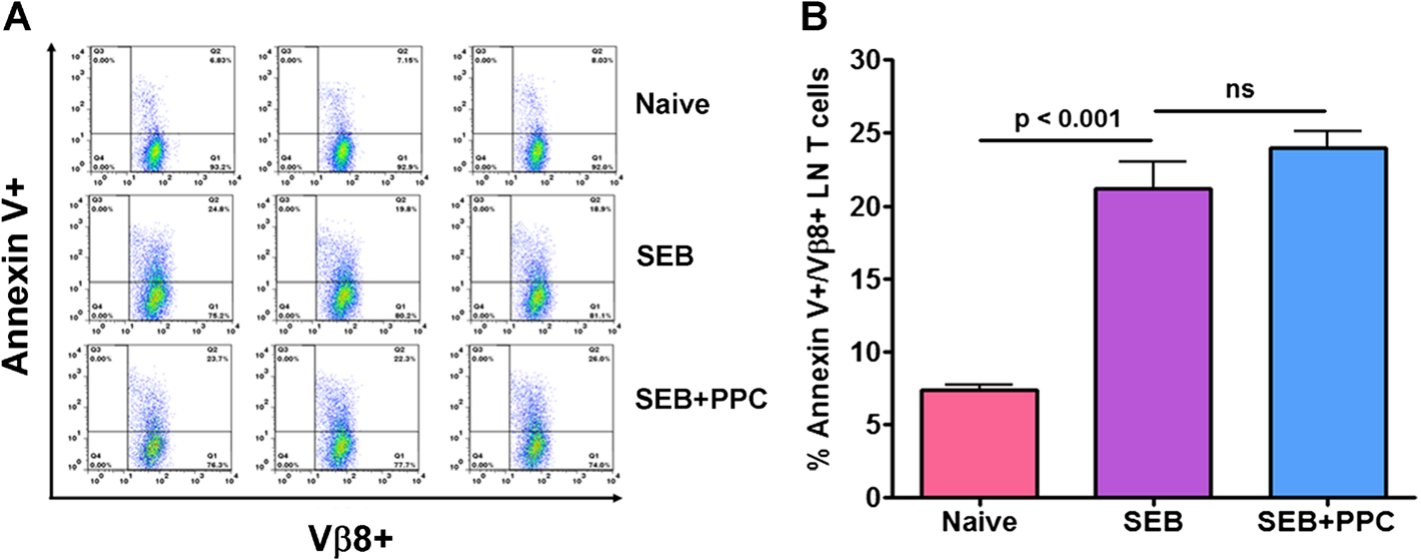
**Detection of early apoptosis in the lymph node Vβ8**^**+ **^**T cells 48 h post injection of SEB.** Balb/c mice (n = 3 per group) were treated as described in the Materials and Methods. The cells were stained for the expression of CD3, Vβ8 and Annexin V and then evaluated by flow cytometry. **(A)** The scatter plots of the Vβ8^+^/Annexin V^+^ T cells from the lymph nodes of the individual mice are shown. **(B)** The percent of Annexin V^+^ cells in the CD3^+^/Vβ8^+^ population of lymph nodes cells is shown. Error bars represent standard deviation. The label (ns) indicates no significant difference between the two groups and a p value > 0.05.

When the lymph node Vβ8^+^ T cells were examined by Annexin V staining, an early sign of apoptosis, there was revealed to be a significant difference between the Naïve and SEB treated groups (Figure 8, p < 0.001) demonstrating the SEB-associated induction of apoptosis. However, there was no difference (p > 0.05) in the levels of apoptosis in the cells isolated from the SEB or SEB + PPC groups of mice. This again suggests that PPC does not play a major role in suppressing the apoptosis associated with the contraction phase of the primary T cell response to SEB.

## Discussion

The report by Burrows et al. [3] describes the ability of orally administered PPC to consistently double the number of antigen-specific CD8^+^/IFNγ^+^ T cells detected during secondary T cell responses in mice that had been immunized with either a DNA, protein or dendritic cell vaccine. All of these T cell responses were measured at times long after the primary response had waned and memory cells had been established [3]. In order to determine if PPC’s activity is specific for the secondary response, we sought to examine its effect on the development of a primary T cell response. To do so, we utilized the well-established SEB model of T cell activation.

When SEB is presented by MHC II bearing APC to Vβ8^+^ T cells, the T cells become activated, begin proliferating, and undergo differentiation. This takes place without the need for antigen processing by the APC. When we examined the effects of oral PPC on the SEB-mediated Vβ8^+^ T cell expansion we detected significantly more CD4^+^ and CD8^+^ Vβ8^+^ T cells in the lymph nodes of mice that had been treated with SEB + PPC. In fact, we again observed a doubling of the number of CD4^+^/Vβ8^+^ and CD8^+^/Vβ8^+^ T cells in the lymph nodes of PPC treated mice (Figure 2). Interestingly, treatment of mice with oral PPC alone appeared to have no effect on T cell expansion (Figure 2). These observations suggest that the PPC effect we previously observed during the secondary T cell response is likely to due to PPC’s ability to affect the primary response.

By 96 h post SEB injection, when the activated T cells are in the midst of contraction, the CD4^+^/Vβ8^+^ T cells in the SEB treated mice had already contracted to levels lower than those found in the lymph nodes of naive mice while the levels of CD8^+^/Vβ8^+^ T cells in the SEB + PPC treated mice were still significantly higher than those found in the mice treated with SEB alone (Figure 3). This finding of an apparent increased contraction rate in the activated CD4+ T cells has been previously described and was therefore expected [8,9,16]. However, the fact that the resulting enhanced expansion of the SEB + PPC stimulated CD4^+^/Vβ8^+^ T cell population did not lead to an enhanced number of CD4^+^/Vβ8^+^ T cells surviving the contraction is curious (Figure 3A). We believe that this observation could be explained by the findings of Liu and Janeway [17], Noble et al. [18], Florido et al. [19], and Atsumi et al. [20]. Their reports describe a strong link between the production of IFNγ by activated CD8^+^ T cells and the induction of CD4^+^, but not CD8^+^, T cell apoptosis. Therefore, the increase in serum IFNγ levels that we detected (Figure 4) could potentially result in a more extensive induction of apoptosis in the CD4^+^/Vβ8^+^ T cell population.

There have been reports suggesting that the production of reactive oxygen species by activated T cells is responsible for the induction of apoptosis [12], [21]. Specifically, it has been shown that the ROS associated with T cell activation works by down regulating the activity of the anti-apoptosis protein, Bcl-2 [12]. Since levels of Bcl-2 within cells are critical for anti-apoptotic activity, decreasing Bcl-2 sensitizes cells to apoptosis. Antioxidants capable of scavenging the ROS have been shown to be able to prevent the ROS-induced down regulation of Bcl-2 and prevent activation induced apoptosis [12].

In 2003, Philippa Marrack’s laboratory discovered that culturing activated T cells (48 hr post SEB treatment) with the peroxynitrite scavenger, MnTBAP, reversed the ROS-induced decline in Bcl-2. They also demonstrated that the pro-apoptosis protein Bim (Bcl-2 interacting mediator of cell death) is required for SEB-driven T cell death and that it is the ROS-induced down regulation of Bcl-2 that leads to increased levels of free Bim. It is the free Bim that alters the mitochondrial membrane potential and activates the apoptosis pathway [12]. Therefore, suppression of the ROS in activated T cells should lead to the production of enough Bcl-2 to bind to Bim and prevent activation of the apoptosis pathway.

Since PPC contains polyphenolics with antioxidant properties [22], we sought to determine if PPC was affecting the generation of ROS by the SEB-activated T cells. As shown in Figure 5, orally administered PPC was able to reduce the ROS levels in the lymph node CD3^+^ T cell population. When we examined the levels of intracellular Bcl-2 in the Vβ8^+^ T lymph node T cells, we found that PPC had no effect on SEB’s suppression of Bcl-2 expression. The T cells from both the SEB and SEB + PPC treated mice had less Bcl-2 per cell than did the cells from the naive control animals (Figure 6). This suggests that the observed decrease in ROS levels by PPC were probably not significant enough to affect the expression of Bcl-2. When the CD3^+^/Vβ8^-^ T cells (cells that should not be activated by SEB) were examined there was found to be no difference between the intracellular levels of Bcl-2 in the lymph nodes from the Naive mice and the SEB or SEB + PPC groups (Figure 6C). The lack of an effect by SEB on the CD3^+^/Vβ8^-^ cells demonstrates the specificity of SEB for T cells expressing the Vβ8^+^ variant of the T cell receptor.

The reduction of intracellular Bcl-2 protein levels detected in the SEB and SEB + PPC groups was paralleled by changes in the mRNA levels for Bcl-2 (Figure 7A). In the same cells we found that while SEB suppressed expression of the anti-apoptotic gene, Bcl-2, it enhanced expression of the pro-apoptosis gene, Bim, by more than 3 fold (Figure 7A). These results are in agreement with those of Hildeman et al. [11] and indicate that activation induced apoptosis had already begun to occur 48 h after injection of the SEB.

Activation of cell apoptosis at the 48 h time point was verified by Annexin V staining. Cells undergoing apoptosis can be identified by their ability to bind fluorescently labeled Annexin V. When compared with the T cells from the Naive mice, SEB significantly enhanced the percentage of Annexin V positive Vβ8^+^ T cells (p < 0.001). Administration of PPC to the SEB treated mice failed to alter SEB’s ability to enhance the expression of Annexin V (Figure 8). When combined with the other indicators of activation induced cell death, it appears that PPC has no significant effect on the contraction phase of the primary T cell response.

## Conclusions

PPC’s ability to double the number the antigen-specific CD8^+^ T cells detected in a secondary immune response following immunization with either DNA, protein, or dendritic cell vaccines appears to be associated with enhancement of the proliferative phase of the primary T cell response. Previous reports from our laboratory demonstrated that the *in vitro* exposure of human PBMC [4] and murine bone marrow cells [5] to PPC enhances the production and maturation of dendritic cells. If PPC is able to similarly affect dendritic cells *in vivo* it is possible that this could result in enhanced activation and proliferation of the SEB-specific T cells. Another possibility is that PPC could be enhancing dendritic cell migration to the lymph nodes. The presence of an increased number of dendritic cells would allow for the presentation of SEB to a larger number of T cells. Since not all Vβ8^+^ T cells proliferate in response to an SEB injection [23], an increase in the number of dendritic cells presenting SEB in the lymph nodes could result in an increased number of activated T cells. In fact, preliminary results have detected an increase in the number of CD11c^+^ cells in the lymph nodes of mice treated with SEB + PPC (data not shown). Further studies are needed to identify the mechanism(s) by which PPC enhances the proliferative phase of a primary T cell response.

## Competing interests

Authors AT and WGB are listed as inventors on patents related to the production of PPC and hold stock in the company (Allera Health Products, Inc) selling the commercial product, Immune Extra™.

## Authors’ contributions

Authors AT and WGB designed the experiments and interpreted the experimental results. WGB and KNH performed all animal work, flow cytometery, gene expression and ELISA assays. All authors contributed to the manuscript preparation and approved its submission.

## Pre-publication history

The pre-publication history for this paper can be accessed here:

http://www.biomedcentral.com/1472-6882/14/163/prepub
